# The effect of radioiodine treatment on the characteristics of TRAb in Graves’ disease

**DOI:** 10.1186/s12902-021-00905-4

**Published:** 2021-11-30

**Authors:** Ya Fang, Wen-Hua Du, Cao-Xu Zhang, Shuang-Xia Zhao, Huai-Dong Song, Guan-Qi Gao, Mei Dong

**Affiliations:** 1 Department of Molecular Diagnostics & Endocrinology, The Core Laboratory in Medical Center of Clinical Research, Shanghai Ninth People’s Hospital, State Key Laboratory of Medical Genomics, Shanghai Jiao Tong University School of Medicine, 200011 Shanghai, China; 2 Department of Endocrinology, Linyi People’s Hospital, Linyi, China

## Abstract

**Background:**

Graves’ disease (GD) is one of the most common autoimmune thyroid diseases (AITDs) in humans, and thyrotropin receptor antibody (TRAb) is a characterized autoantibody in GD. The use of radioactive iodine therapy (RAI) for GD treatment is increasing.

**Objectives:**

We studied the biological properties of TRAb and evaluated the effect of RAI therapy on TRAb in GD patients.

**Methods:**

In total, 225 patients (22 onset GD patients without ^131^I therapy, 203 GD patients treated with ^131^I therapy) and 20 healthy individuals as normal controls were included in this study. Clinical assessments were performed, and we examined *in vitro* the biological properties of TRAb in the 22 onset GD patients and 20 controls as well as 84 GD patients with ^131^I therapy.

**Results:**

Serum TRAb and thyroid peroxidase antibody (TPOAb) levels increased in the initial year of RAI treatment, and both antibodies decreased gradually after one year. After 5 years from radioiodine treatment, TRAb and TPOAb levels decreased in 88% and 65% of GD patients, respectively. The proportion of patients positive for thyroid-stimulatory antibody (TSAb) was significantly higher in the 7–12-month group, and thyroid-blocking antibody (TBAb) levels were elevated after one year in half of the patients who received ^131^I treatment.

**Conclusions:**

Treatment of GD patients with radioiodine increased TPOAb and TRAb (their main biological properties were TSAbs) within the first year after therapy, and the main biological properties of elevated TRAb were TBAbs after 1 year.

## Background

Autoimmune thyroid diseases (AITDs), which primarily include Graves’ disease (GD) and Hashimoto’s thyroiditis (HT), are common organ-specific autoimmune diseases with varying severity and intractability [1]. AITDs are characterized by lymphocytic infiltration into the thyroid and the production of autoantibodies to thyroid-specific antigens, such as thyrotropin receptor (TSHR), thyroid peroxidase (TPO) and thyroglobulin (Tg) [2–4]. The presence of thyrotropin receptor (TSHR) autoantibody (TRAb) is used in the serological diagnosis of GD [5]. TRAb has different biological properties, including thyroid-stimulatory antibodies (TSAbs), thyroid-blocking antibodies (TBAbs) and neutral TSH receptor antibodies [6, 7]. Although a positive TRAb test result suggests the presence of TSAb or TBAb, it is reasonable to presume that a positive result in a patient with hyperthyroidism is due to TSAb. TSAb behaves like TSH and stimulates the synthesis of thyroid hormone by binding to TSHR, which leads to hyperthyroidism [8]. TSAb also causes diffuse, hypervascular goiter in many GD patients. However, some HT patients are TRAb positive, showing hypothyroidism rather than hyperthyroidism [9].

Radioactive iodine therapy (RAI) is a beneficial choice for the treatment of patients with GD in some countries [10] because it is easy to administer, relatively inexpensive, safe and highly effective [11]. However, hypothyroidism is the main side effect of RAI treatment in patients with hyperthyroidism. TRAb decreases in some GD patients after RAI but increases in other patients. Such transient increases in TRAb levels in GD patients after the first several months from ^131^I treatment might be mediated by the release of thyroid antigens from damaged thyrocytes [12, 13]. Previous reports have found that GD patients with a significant increase in TSAb after 6 months from ^131^I treatment develop hypothyroidism later [14]. This increase in TRAb can persist for many years in a few GD patients after ^131^I treatment, indicating that other factors that induce the production of TRAb exist.

The biological activities of TRAb may be assessed *in vitro* using cells transfected with TSH receptors that distinguish stimulating and blocking antibodies against TSHR [7]. The present study investigated patients with GD who received RAI and analyzed clinical changes in these patients after RAI therapy. We evaluated the biological properties of their TRAbs and assessed factors that may modulate the response. We also explored differences in the biological properties of TRAbs in the onset GD patients.

## Subjects and methods

### Subjects

A total of 225 unrelated individuals with GD were recruited from Linyi People’s Hospital and the Ninth People’s Hospital Affiliated to Shanghai Jiao Tong University School of Medicine from May 2018 until August 2019. Among these 225 GD patients, 203 were treated with ^131^I therapy; the other 22 patients were diagnosed with GD onset and were not treated with ^131^I therapy. The control group was composed of 20 unrelated healthy subjects from the same geographic region who were screened for thyroid autoimmune antibody (TRAb, TGAb and TPOAb) negative and had no family history of thyroid disease (age range from 26 to 58 years old and 9 females and 11 males). All of the subjects provided informed written consent, and the local ethics committee (the Ninth People’s Hospital Affiliated to Shanghai Jiao Tong University School of Medicine) approved the project, which was performed in accordance with the ethical standards of the Declaration of Helsinki (2013 version) and its later amendments or comparable ethical standards. All cases conformed to the diagnostic and treatment criteria of thyroid disease in China (2007), which were compiled by experts from the Endocrinology Branch of the Chinese Medical Association [15, 16]. Diagnosis of GD was based on the principles described in our previous reports: clinical and biochemical manifestations of hyperthyroidism, diffuse goiter and at least one of the following phenotypes: positive TSH receptor antibody tests; diffusely increased ^131^I (iodine-131) uptake in the thyroid gland or exophthalmos [17–19].

For the 203 GD patients treated with ^131^I, the following parameters were registered: age (43.98±12.39 years), female/male sex (*n *= 172/31), duration of GD (1.97±3.66 years), and treatment with corticosteroids (*n *= 13). The ^131^I dose (mCi) was equal to the thyroid mass (g) multiplied by the ^131^I dose per gram thyroid tissue (µCi/g) and divided by the 24-h maximal ^131^I uptake rate (8.59±3.23 mCi). The 22 untreated GD patients had a female:male ratio of 19:3, with an age range of 40.09±13.71 years. The control group of 20 healthy individuals had a female:male ratio of 9:11, with an age range of 42.20±15.87 years, and had no history of autoimmune thyroid disease and normal values for TSH, FT3, FT4, TRAb and TPOAb. For the TSAb and TBAb subgroups, patients were selected randomly to ensure that those with different TRAb levels were chosen. Sera from subjects were collected by centrifuging whole blood at 2000 rpm for 10 min and then heated at 56 ℃ for 30 min to remove complement. The samples were aliquoted and stored at -80 ℃.

### Assays

Serum FT3, FT4, TSH and TPOAb were detected using an Elecsys 2010 Chemiluminescence Immunoassay Analyzer (Roche, USA) and special auxiliary reagents (Roche, USA) according to the manufacturer’s instructions. Serum TRAb was measured using the radiation receptor method with an assay kit (Union-med, China) [20]; the detection limit was 0.3 IU/L. The normal ranges of these parameters were FT3 (3.5–6.5 pmol/L), FT4 (11.5–22.7 pmol/L), TSH (0.55–4.78 mIU/L), TPOAb (0–60 IU/mL), and TRAb (0–1.75 IU/L).

TSHR-expressing Chinese hamster ovary (CHO) cells were incubated with test serum or control serum. TSAb and TBAb levels were determined using a functional bioassay based on induction or inhibition of cAMP production. The cAMP concentration in transfected cells was measured using a cAMP assay kit (R&D Systems assay, USA) [21]. Briefly, 4 × 10^4^ stably transfected TSHR-CHO cells were seeded in 48-well plates. On the following day, the medium was removed, and the cells were incubated in Krebs-Ringer phosphate buffer (KRPB) for 15 min at 37 °C. For stimulation experiments, the KRPB buffer was removed, and the cells were incubated in 100 µl patient or control serum for another 2 h at 37 °C. In blocking experiments, the KRPB buffer was removed, and the cells were incubated with 100 µl patient or control serum with 1 IU/L bovine TSH (bTSH; Sigma Aldrich, USA) for 2 h at 37 °C. The cells were washed twice with cold PBS, lysates were prepared, and the cAMP concentration in the cells was measured as described in the instructions for the cAMP assay kit.

Activities of TSAb and TBAb were calculated as follows: TSAb activity (percent) =100 × (cAMP patient/cAMP control) and TBAb activity (percent) = 100 × [1- (cAMP patient-bTSH/cAMP control-bTSH)].

### Statistical analysis

Data were analyzed using SPSS 19.0 statistical software, and the results are presented as means ± standard deviation. The normality of variables was estimated using the Kolmogorov–Smirnov test. One-way ANOVA was applied to detect multivariate significance. The Mann–Whitney U test, Welch’s t test and Wilcoxon test were employed to compare significant differences between two groups, and *p *< 0.05 was considered a statistically significant difference. Fold changes in antibodies were determined: a fold change of 1.1 or greater was considered an increase, an inverse ratio of 1.1 or greater was considered a decrease, and the remaining ratios were defined as unchanged.

## Results

### Clinical characteristics of patients

Our study enrolled 20 healthy individual controls, 22 onset GD patients without ^131^I therapy and 203 GD patients who received ^131^I therapy. Paired serum samples were collected from 203 GD patients before and after ^131^I therapy. The features of their thyroid function are provided in Tables 1 and 2. Among the 203 GD patients administered ^131^I, 78 received antithyroid drugs before the therapy. After radioiodine treatment, there were no significant differences between the GD patients treated with or without drugs with regard to the following characteristics: age, FT3, FT4, TSH, TPOAb and TRAb (data not shown). After ^131^I therapy, the overall average TRAb and TPOAb levels of the GD patients decreased significantly compared to the levels before RAI therapy (Table 1). To assess RAI prognosis more accurately, the 203 GD patients were divided into 4 groups based on the treatment course after ^131^I therapy: 22 after 1–6 months from ^131^I treatment, 28 after 7–12 months from ^131^I treatment, 105 after 13–60 months from ^131^I treatment and 48 after more than 60 months from ^131^I treatment. Among the four groups, we found no significant differences in the distribution of age, levels of FT4, TPOAb, or TRAb, ^131^I dose or 24-h maximal ^131^I uptake rate (%) before ^131^I therapy. However, the TSH level baseline was the lowest in those after 7–12 months from ^131^I therapy, compare to the other three groups (Table 2).

**Table 1:** The clinical characteristics of patients with GD and normal controls

Variable	Control group	Onset GD without ^131^I therapy	GD before ^131^I therapy	GD after ^131^I therapy	Normal range
No.	20	22	203		
Age(y)	42.20±15.87	40.09±13.71	43.98±12.39		
Gender(M/F)	11/9	3/19	31/172		
FT3(pmol/L)	4.70±1.39	21.17±9.62	20.77±8.85	4.46±1.32	3.5-6.5
FT4(pmol/L)	14.69±4.44	64.48±44.15	56.77±34.57	15.27±7.47	11.5-22.7
TSH(mIU/L)	3.19±3.09	0.002±0.001	0.09±0.75	12.95±24.65	0.55-4.78
TRAb(IU/L)	0.65±0.48	11.28±10.17	16.95±12.47^***^	10.14±13.30	0-1.75
TPOAb(IU/mL)	29.44±4.30	644.90±625.50	839.35±567.10^###^	678.13±573.41	0-60

Data are expressed as mean±standard deviation according to the distribution

*M* male, *F* female, *GD* Graves’ disease

^***^p < 0.001 represents TRAb levels of GD before ^131^I therapy versus GD after ^131^I therapy

^###^*p *< 0.001 represents TPOAb levels of GD before ^131^I therapy versus GD after ^131^I therapy

**Table 2:** Comparison of clinical data before 131I therapy between groups

Group	1-6 months	7-12 months	13-60 months	>60 months
Gender(M/F)	6/16	6/22	12/93	7/41
Age(y)	45.50±11.70	38.86±11.96	44.67±12.64	44.77±11.67
FT3(pmol/L)	21.18±8.86	25.05±6.35^*^	18.87±9.05^*^	22.18±8.38
FT4(pmol/L)	62.64±37.24	64.02±35.42	52.18±30.28	63.41±37.87
TSH(mIU/L)	0.06±0.25	0.004±0.003^#^	0.06±0.36	0.01±0.017
TPOAb(IU/mL)	859.05±575.77	899.25±550.87	835.48±562.10	802.49±574.35
TRAb(IU/L)	15.76±12.15	15.18±12.55	16.97±12.11	18.48±13.00
^131^I dose(mCi)	8.14±2.32	9.59±2.86	8.74±3.53	7.84±2.86
24 h maximal ^131^I uptake rate(%)	73.89±9.55	68.91±10.09	72.24±12.92	75.85±12.42

Data are expressed as mean ± standard deviation according to the distribution.

^*^p <0.05 represents 7-12 months group versus 13-60 months group

^#^*p* <0.05 represents 7-12 months group versus the other three groups

### The natural course of TRAb and TPOAb in GD patients after treatment with radioiodine

**Fig. 1:**
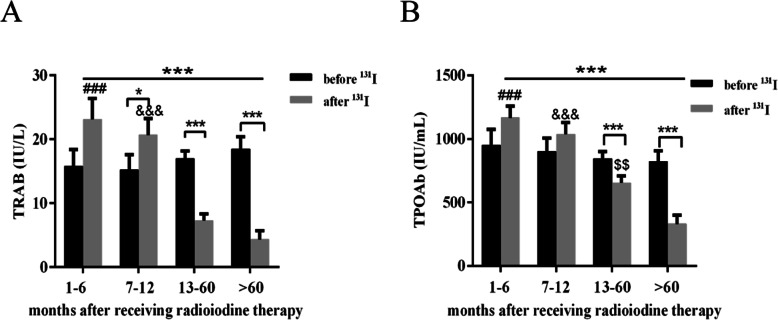
Time course changes in antibodies after ^131^I therapy. **A** and **B** Show time course changes in TRAb and TPOAb in Graves’ disease patients before and after ^131^I therapy. *denotes *p *< 0.05, ***denotes *p *< 0.001, ^###^*p *< 0.001 represents the 1–6 months group versus 13–60 months group and >60 months group, ^&&&^*p *< 0.001 represents the 7–12 months group versus 13–60 months group and >60 months group, ^$$^*p *< 0.01 represents the 13–60 months group versus >60 months group

**Fig. 2:**
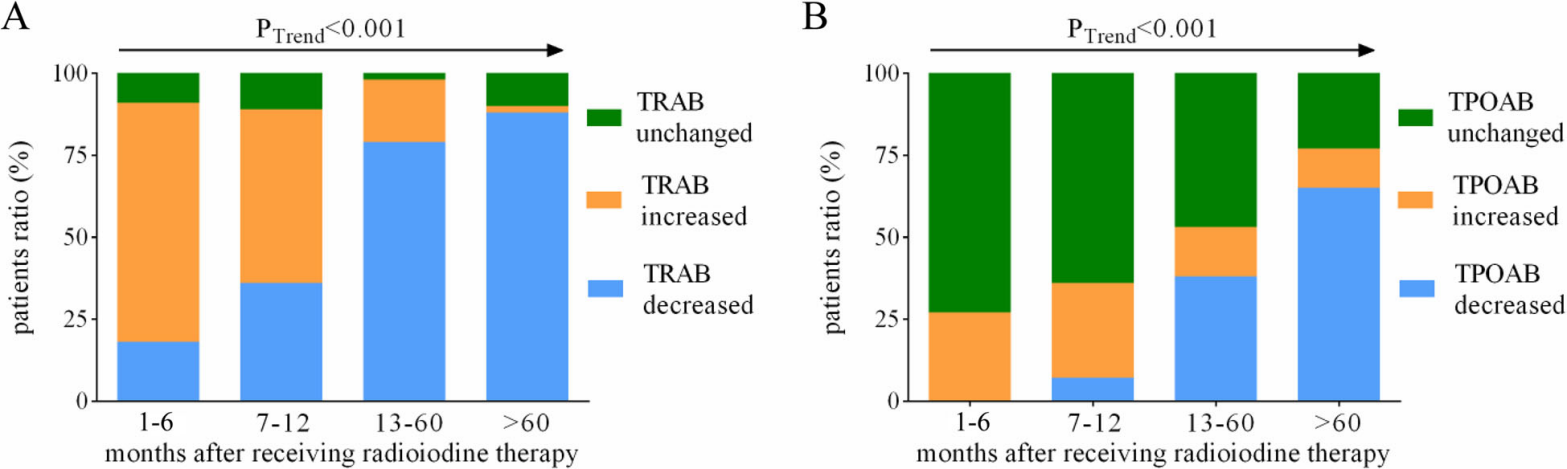
Changes in TRAb and TPOAb at different times after ^131^I treatment. This picture shows the time course discrepancy of the GD patient ratio with different TRAb changes (**A**) and TPOAb changes (**B**) after RAI therapy. The p value was obtained by the Trend Test

To further investigate the natural course of TRAb and TPOAb in GD patients after treatment with ^131^I therapy, we compared serum levels of TRAb and TPOAb before and after ^131^I radiotherapy in the above four groups. No significant differences in TRAb and TPOAb levels emerged comparing baseline data with those after 1-6 months from radioiodine treatment (Fig. 1A, B). After 7–12 months from ^131^I therapy, serum levels of TRAb increased significantly compared to data before radioiodine therapy (Fig. 1A). In the group of GD patients after 7–12 months from ^131^I therapy, no significant difference in TPOAb was detected compared with data before treatment with ^131^I (Fig. 1B). Notably, serum levels of TPOAb and TRAb in the GD patients after 13–60 months or over 60 months from ^131^I therapy were significantly lower than those after 1-6 months from ^131^I therapy (Fig. 1A, B). We also found that levels of TRAb in 88% and TPOAb in 65% of GD patients decreased by more than 1.1 times after 5 years from they received radioiodine therapy (Table 3; Fig. 2).

**Table 3:** Changes of TRAb and TPOAb at different time after 131I treatment

	GD patients with decreased antibodies		GD patients with increased antibodies		GD patients with unchanged antibodies	
	TRAb^***^	TPOAb^###^	TRAb	TPOAb	TRAb	TPOAb
1-6 m	18% (4/22)	0 (0/22)	73% (16/22)	27% (6/22)	9% (2/22)	73% (16/22)
7-12 m	36% (10/28)	7% (2/28)	53% (15/28)	29% (8/28)	11% (3/28)	64% (18/28)
13-60 m	79% (83/105)	38% (40/105)	19% (20/105)	15% (16/105)	2% (2/105)	47% (49/105)
>60 m	88% (42/48)	65% (31/48)	2% (1/48)	12% (6/48)	10% (5/48)	23% (11/48)

TRAb or TPOAb increased: TRAb of ^131^I therapy after/before ≥1.1

TRAb or TPOAb decreased: TRAb of 131I therapy before/after ≥1.1

^***^p <0.001 represents changes of TRAb comes from Trend Test

^###^*p* <0.001 represents changes of TPOAb comes from Trend Test

### Biological characterization of TRAb

**Fig. 3:**
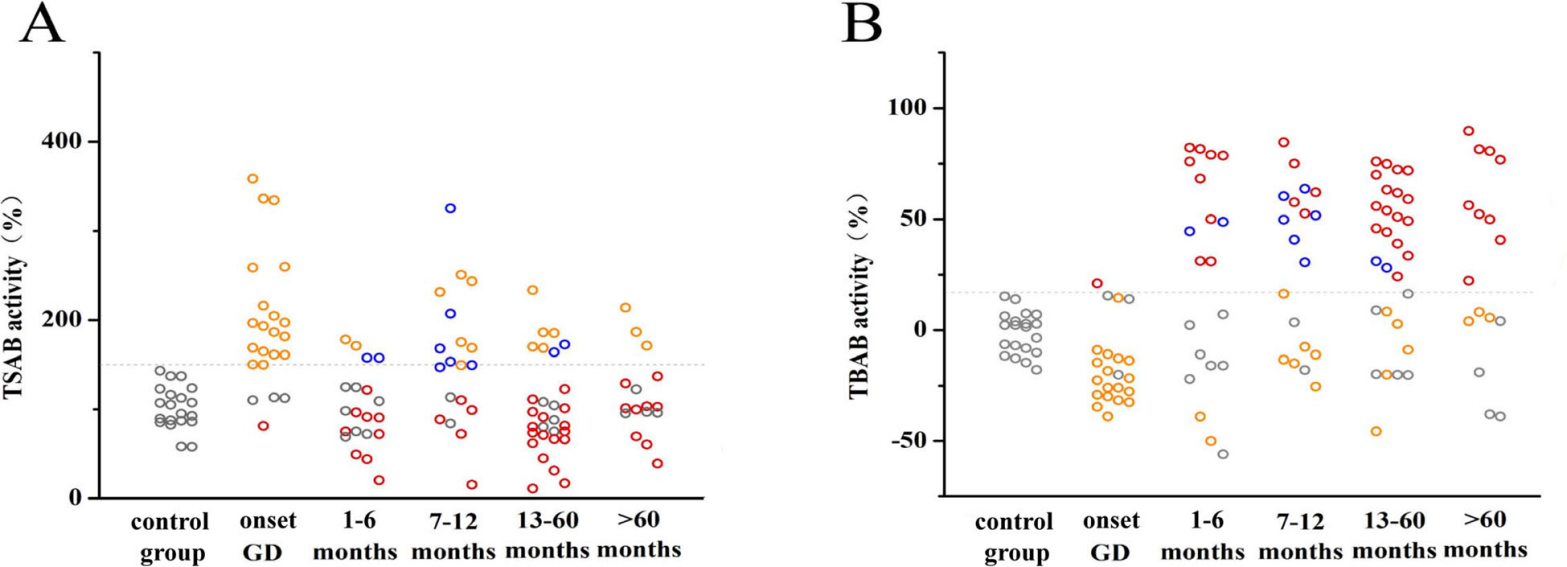
Bioactivity of TRAb in GD patients treated with ^131^I therapy. TRAb bioactivity was measured in 22 onset Graves’ disease patients without radioiodine therapy and 84 Graves’ disease patients treated with radioiodine therapy (1–6 months group [*n *= 20], 7–12 months group [*n *= 19], 13–60 months group [*n *= 29], >60 months group [*n *= 16]). **A** and **B** Show TSAb and TBAb activities in these patients, respectively. The gray circle represents patients with normal levels of TSAb and TBAb; the yellow circle represents patients with high TSAb; the red circle represents patients with high TBAb; the blue circle represents patients with high TSAb and TBAb simultaneously

To further investigate changes in TRAb after radioiodine treatment, we performed cell experiments to analyze the biological properties of TRAb, with TRAb bioactivity being measured in 20 controls, 22 onset GD patients without radioiodine therapy and 84 patients treated with radioiodine therapy (1–6 months group [*n *= 20], 7–12 months group [*n *= 19], 13–60 months group [*n *= 29], >60 months group [*n *= 16]), who were randomly chosen with different TRAb levels to ensure the highest coverage. Most patients in the onset GD group had detectable TSAbs (82%, 18/22), whereas only 4.5% of patients had TBAbs (1/22). The proportion of patients with TSAbs after 1-6 months from ^131^I therapy decreased significantly, but the proportion of those with TBAbs increased (20% vs. 55%). 10% of patients showed both TSAbs and TBAbs. The proportion of patients with TSAbs after 7–12 months from ^131^I therapy increased (63%, 12/19); the proportion with TBAbs increased slightly compared with those after 1-6 months from ^131^I therapy (58%, 11/19). However, the proportion of patients showing both TSAbs and TBAbs increased (32%, 6/19). Among those after 13–60 months from ^131^I therapy, the proportion with TSAbs decreased (24%, 7/29), and 7% of patients showed TSAbs and TBABs simultaneously. Meanwhile, the proportion of TBAb-positive patients was increased in this group (65%, 19/29). The rate of TSAb-positive patients after over 60 months from receiving ^131^I therapy exhibited a greater decrease than the other groups (19%, 3/16), and the proportion of patients with TBAbs was 56% (9/16). (Figure 3A, B). Levels of TSAb and TBAb were 58–143% and -18–15%, respectively. We defined above the mean +2 SD as positive (TSAB ≥150%, TBAB ≥17%).

### Correlation of TSAb/TBAb and thyroid function after ^131^I therapy

**Fig. 4:**
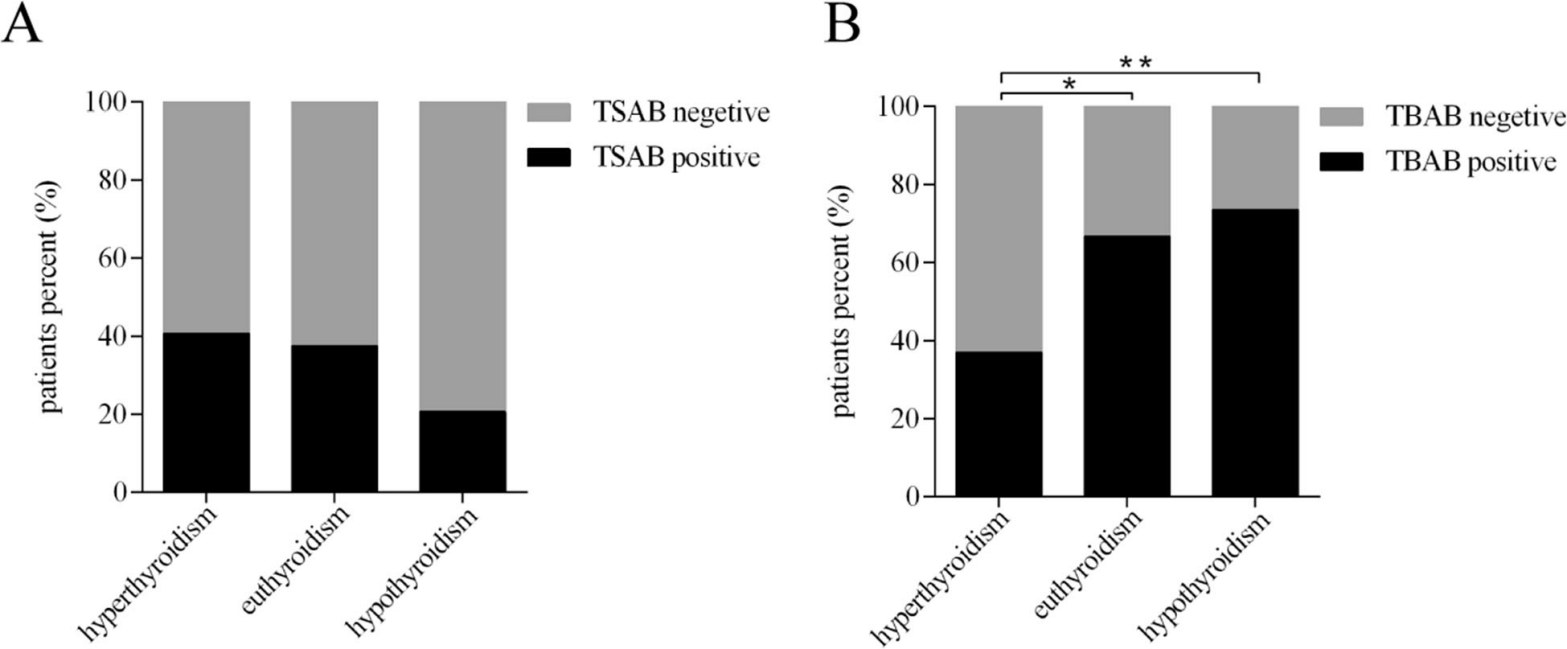
Proportion of TSAb- or TBAb-positive patients among GD patients with different thyroid functions after ^131^I therapy. **A** and **B** Show ratios of TSAb-positive and TBAb-positive patients with Graves’ disease with different thyroid functions after ^131^I therapy. *denotes *p *< 0.01, **denotes *p *< 0.001

We further investigated the correlation between thyroid function and TSAb/TBAb in GD patients after ^131^I therapy. The proportions of TSAb positivity in patients with hyperthyroidism, euthyroidism and hypothyroidism were 40.7% (11/27), 37.5% (9/24) and 20.5% (7/34), respectively. Although the proportion of TSAb-positive GD patients with hyperthyroidism after ^131^I therapy was slightly higher, no significant differences were found among the groups (Fig. 4A). The proportions of TBAb-positive GD patients after ^131^I therapy with hyperthyroidism, euthyroidism and hypothyroidism were 37% (10/27), 66.7% (16/24) and 73.5% (25/34), respectively. Interestingly, the proportion of TBAb-positive GD patients with hypothyroidism or euthyroidism after ^131^I therapy was higher than that in GD patients with hyperthyroidism (73.5% vs. 37%, *p *< 0.001; 66.7% vs. 37%, *p *< 0.01, respectively) (Fig. 4B).

## Discussion

GD and HT are two main types of AITDs with different physiopathologies. TRAb and TPOAb are characterized autoantibodies in GD [22]. TRAbs exhibit three different biological properties, including TSAbs, TBAbs and neutral TSH receptor antibodies. TSAbs are responsible for GD hyperthyroidism, whereas TBAbs are sometimes responsible for a pattern of hypothyroidism [23]. ^131^I therapy is increasingly used as a first-line treatment for hyperthyroidism in GD [20], and previous studies have reported that patients with GD treated with a dose of ^131^I can develop hypothyroidism. Although the traditional view considers GD and HT to be two separate diseases, the present view is that these conditions may be opposite ends of the spectrum of one disease. Indeed, a few researchers have reported a sequential phenotypic conversion from GD to HT or *vice versa* [24–26]. The underlying mechanism is not clear but may be determined by the following factors: TSAb and TBAb activity, responsiveness of the thyroid gland to TSAb or TBAb, and changes in the thyroid gland [23]. TSAbs and TBAbs are antibodies against TSHR, but the antigens are different. The uncleaved, single-chain TSHR polypeptide consists of 764 amino acids (including a 21-residue signal peptide absent in the mature protein). After expression on the thyrocyte cell surface, TSHR undergoes cleavage within the “hinge” region at two or more sites. Loss of the C-peptide-like region leads to an extracellular A-subunit linked by disulfide bonds to the B-subunit, which comprises the remainder of the hinge region, transmembrane, and cytoplasmic tail. Some A-subunits are shed. Substantial evidence suggests that the shed A subunit, rather than the TSH holoreceptor, is more important in the induction or affinity maturation of TSAbs that cause GD [27, 28]. In contrast, the holoreceptor is a much more potent immunogen for TBAbs. In the present study, we found a period in which TSAbs and TBAbs exist simultaneously in some patients after ^131^I therapy; the proportion of TSAbs decreased while that of TBAbs increased. Therefore, it is reasonable to presume that ^131^I therapy causes thyrocyte damage in GD patients, which can lead to the release of thyroid antigens, including the holoreceptor of TSHR and other antigens, further triggering the autoimmune response to induce the production of TBAbs and other autoantibodies against thyroid antigens [28]. Interestingly, McLachlan et al. found that by depletion of T regulatory cells (Treg) with anti-CD25 before TSHR-Ad immunization, the immunization with TSHR-Ad induced extensive thyroid lymphocytic infiltration and hypothyroidism in human TSHR A subunit-expressing transgenic mice (named Lo-expressor TSHR A subunit transgenic mice) but not in these mice without Treg depletion. Moreover, autoantibodies against mTPO and mTG were also induced by TSHR-Ad immunization in Lo-expressor TSHR A subunit transgenic mice after depletion of Treg with anti-CD25, similar to our findings in GD patients after RAI therapy. Given that the mice had thyroid lymphocytic infiltration and hypothyroidism, it will be interesting to investigate the biological characterization of TRAb in the future in Lo-expressor TSHR A-subunit transgenic mice depleted of Treg cells with anti-CD25 and immunized with TSHR-Ad [29]. Takasu et al. found that all of the HT patients with hypothyroidism (*n *= 43) exhibited TBAb positivity in European patients [30]. A previous study reported that HT patients could spontaneously recover from hypothyroidism, with TBAb disappearance [31]. Therefore, the hypothyroidism in these patients may be attributed to TBAb. The data suggested that the increase of TBAb might play a role in the process of hypothyroidism in GD patients after ^131^I therapy.

In addition to genetic and environmental factors, treatment for hyperthyroidism also affects GD activity [32, 33]. A prospective randomized study compared antithyroid drugs, thyroidectomy and radioiodine and found that the two former treatments resulted in a continuous decrease in TRAb but that radioiodine resulted in an increase in TRAb after 3 months [34]. Lindgren et al. compared TRAb, TPOAb and TGAb before and 3 months after ^131^I therapy and found that radioiodine elicited an increase in these antibodies, though not in all GD patients [35]. In the present study, most patients exhibited increases in TRAb in the first year from ^131^I therapy, and most exhibited decreases in TRAb after 1 year from therapy. Some patients showed increases in TPOAb during the first year from therapy, but the proportion was much lower than that of patients with increased TRAb. Further investigation revealed a decrease in TSAb-positive patients in the 1-6 month group and an increase in TSAb-positive patients in the 7–12-month group. After one year, the TSAbs decreased gradually. However, TBAb was elicited after ^131^I therapy, and the level increased in the first 5 years. After one year from radioiodine treatment, most patients were TBAb positive. Following ^131^I therapy, thyroid secretion declined gradually over weeks to months. Approximately 50–70% of patients become euthyroid within 6 to 8 weeks, with a concomitant marked reduction in thyroid size [36]. In general, when receiving a calculated dose or a fixed dose in the 10 to 15 mCi (370–555 MBq) range, 80–90% of patients ultimately become euthyroid or hypothyroid after one dose of ^131^I; 10 to 20% require a second dose, and only rare need an additional dose [37]. Our study found that TSAb positivity was more likely to be present in those patients after 7–12 months from radioiodine therapy. Therefore, we suggest that the first treatment dose of radioiodine for GD patients should not be high and that slightly higher thyroid function may be tolerated in patients in the first year from receiving ^131^I treatment. For patients exhibiting high T4 after the first ^131^I dose, anti-thyroid drugs may be more beneficial than the second dose of ^131^I in the first year from ^131^I treatment. Notably, we observed a positive relationship between the ^131^I dose and development of hypothyroidism within the first year of therapy. However, the incidence of hypothyroidism beyond 1 year was approximately 2–3% annually and seemed largely independent of the ^131^I dose. We analyzed this phenomenon, which may be caused by the continuous existence of TBAb, and the underlying mechanism must be further investigated. Although there is no established teratogenic risk with radioactive iodine, it has been suggested that conception should be deferred for at least 4 months after therapy [36]. In fact, previous reports have suggested transplacental passage of TSAbs from mother to fetus as the cause of neonatal thyroid dysfunction [38, 39]. Our data suggest that TSAb levels should be detected in women of childbearing age after 7–12 months from radioiodine treatment to decide the appropriate time to conceive.

## Conclusions

In conclusion, we found that treatment with radioiodine elicited an increase in TSAb and TPOAb in the first year after therapy. As there was a decline after 1 year, the dose of ^131^I should not be high, and TSAb levels should be detected in women of childbearing age after 7–12 months from radioiodine treatment for decision-making regarding conception. Our study detected a trend that after ^131^I therapy the proportion of patients with both TSAbs and TBAbs first increased and then decreased; the proportion of patients with TBAbs increased, which suggests that the pathogenic mechanisms of the two AITDs may be opposite ends of the spectrum of one disease.

## Data Availability

The datasets used and/or analyzed during this study are available from the corresponding authors on reasonable request.
